# Improved Detection of Cavernous Sinus Invasion of Pituitary Macroadenomas with Ultra-High-Field 7 T MRI

**DOI:** 10.3390/life13010049

**Published:** 2022-12-24

**Authors:** Felix Eisenhut, Manuel Alexander Schmidt, Michael Buchfelder, Arnd Doerfler, Sven-Martin Schlaffer

**Affiliations:** 1 Department of Neuroradiology, University Hospital Erlangen, Friedrich-Alexander-Universität Erlangen-Nürnberg, Schwabachanlage 6, 91054 Erlangen, Germany; 2 Department of Neurosurgery, University Hospital Erlangen, Friedrich-Alexander-Universität Erlangen-Nürnberg, Schwabachanlage 6, 91054 Erlangen, Germany

**Keywords:** pituitary macroadenoma, ultra-high-field MRI, cavernous sinus infiltration, intraoperative findings

## Abstract

To compare 7 T magnetic resonance imaging (MRI) of pituitary macroadenomas (PMA) with standard MRI and intraoperative findings regarding tumor detection, localization, size, and extension. Patients with suspected pituitary adenoma underwent pre-operative 1.5 T or 3 T and 7 T MRI; 14 patients with a PMA were included. A qualitative (lesion detection, location, cavernous sinus infiltration) and quantitative (lesion size, depth of cavernous sinus infiltration) analysis of 1.5 T, 3 T and 7 T MRI was performed and compared with intraoperative findings. Both 1.5/3 T and 7 T MRI enabled the detection of all PMAs; lesion size determination was equal. 7 T MRI enables more precise assessments of cavernous sinus infiltration of PMA (n_correct 7T_ = 78.6%, n_correct 1.5/3T_ = 64.3%). Ultra-high-field MRI is a reliable imaging modality for evaluation of PMAs providing exact information on lesion location and size. 7 T MRI yielded more accurate information on cavernous sinus infiltration with better agreement with intraoperative findings than standard MRI.

## 1. Introduction

Pituitary adenomas are among the most frequent primary intracranial neoplasms with an estimated prevalence of over 20% in radiologic studies [1], and by far the most common pituitary lesions [2] with an incidence of 3.9 to 7.4 cases per 100,000 [3]. They can be divided either by size in micro- (<1 cm) and macroadenomas (>1 cm) or based on whether these adenomas show hormonal oversecretion causing specific diseases (e.g., acromegaly) or not [4]. In this context, prolactin-secreting adenomas represent the majority of tumors [5], followed by non-functioning adenomas [6]. Although pituitary adenomas are referred to as benign tumors [7], they can show invasive growth behavior with affection and compression of adjacent structures [8], especially if they are macroadenomas.

Due to the complex anatomy of the pituitary gland (PG) and the diminutive size of its associated neoplastic lesions, high-field 3 T magnetic resonance imaging (MRI) with its high spatial resolution is currently the preferred modality for sellar imaging, detection of pituitary adenomas and a possible cavernous sinus (CS) infiltration in the clinical routine [9,10,11,12,13].

Following the introduction of ultra-high-field 7 T MRI scanners with superior spatial resolution and increased signal-to-noise- and contrast-to-noise-ratio [14] into the clinical routine, initial studies focused on the feasibility of 7 T imaging of the sellar region in healthy volunteers [15] and its diagnostic value for detection of microadenomas in Cushing patients compared with 1.5 T MRI [16].

However, not only patients with microadenomas [17], but also patients with pituitary macroadenomas (PMA) could possibly benefit of ultra-high-field MRI—in particular regarding the identification and the extent of a CS infiltration [18,19,20,21]. Especially in secreting adenomas, this infiltration averts the neurosurgical aim of complete adenoma resection and hormonal normalization. As consequence, additive medical or radiotherapeutic procedures are then needed to achieve hormonal normalization. The extent of infiltration of the CS is commonly determined using the Knosp scale [22]. It is generally accepted that in cases of tumor invasion with Knosp 3° or higher, complete tumor resection is nearly impossible and therefore associated with postoperative residual tumor [23,24].

We hypothesize, that 7 T MRI allows a more precise assessment of macroadenoma infiltration of the CS with consequently higher agreement with the intraoperatively determined infiltration grade.

Thus, in this study, we assessed the diagnostic value of 7 T MRI in PMA with a focus on CS infiltration and correlation of radiological with intraoperative findings.

## 2. Materials and Methods

### 2.1. Patients

**Figure 1:**
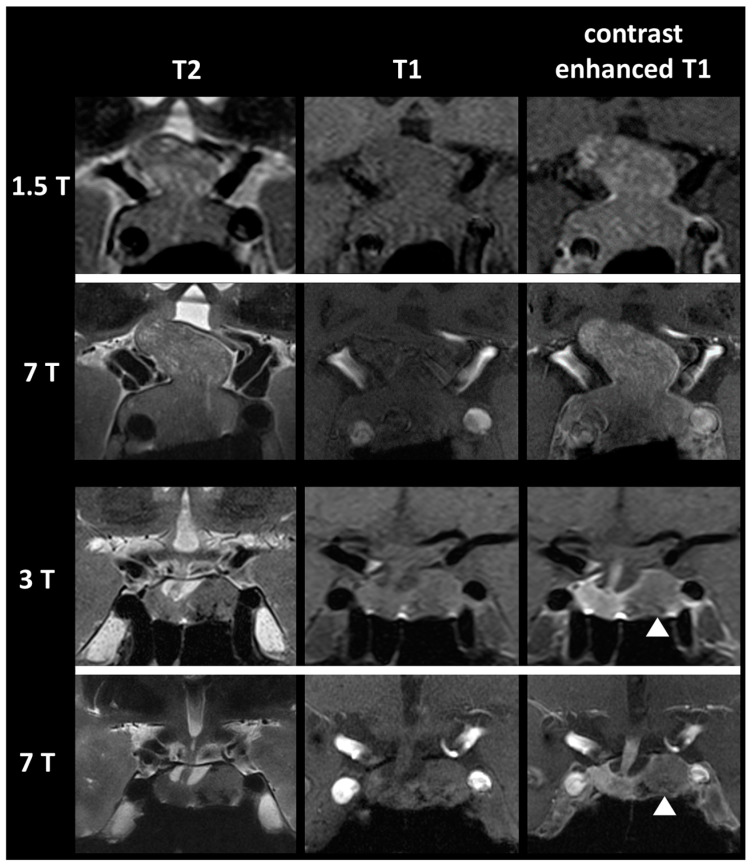
Exemplary 1.5 T, 3 T and 7 T MRI of a pituitary gland macroadenoma in coronal view.

All patients with suspected pituitary tumor based on endocrinological diagnostics and clinical symptoms underwent preoperative 1.5/3 T and 7 T MRI (from October 2017 to November 2019). Patients with pituitary lesions then underwent transsphenoidal resection at the department of neurosurgery. Patients with a histologically proven PMA were included in our analysis. Exemplary 1.5 T, 3 T and 7 T MR images of patients with a PMA are presented in Figure 1.

Written informed consent for MRI was obtained from all patients before all measurements. This study was performed according to the Declaration of Helsinki and the European Guidelines for Good Clinical Practice. Additional ethical review was not required for participation in this analysis in accordance with local legislation (BayKrG Section 27, Paragraph 4) and institutional requirements.

### 2.2. Acquisition and Postprocessing

#### 2.2.1. MRI

1.5 T MRI was performed on a Magnetom Aera, 3 T MRI was performed on a Magnetom Trio Tim, 7 T MRI was performed on a Magnetom Terra (all Siemens Healthineers AG, Erlangen, Germany).

#### 2.2.2. Imaging Protocol and Sequence Details

The MRI examinations performed included a coronal and axial T2-weighted sequence, a coronal T1-weighted sequence, and a coronal and sagittal, contrast-enhanced T1-weighted sequence. For further details, see Table 1.

For contrast-enhanced imaging a quantity of 10 mL 1.0 mmol/mL Gadovist (Bayer AG, Leverkusen, Germany) or 20 mL 0.5 mmol/mL DOTAGRAF (Jenapharm GmbH & Co. KG, Jena, Germany) was intravenously administrated automatically for 1.5/3 T MRI at 3.5 mL/s and manually for 7 T MRI, followed by a 10 mL saline flush.

### 2.3. Data Evaluation

All MRI datasets were analyzed using regular, commercially available, clinical software (syngo.plaza, Siemens Healthineers AG, Erlangen, Germany) in a consensus reading by the authors F.E. (5 years of experience) and M.A.S. (9 years of experience). All readers were blinded to patient data. The assessment was performed separately for 1.5/3 T and 7 T MRI. Patient order was randomized.

#### 2.3.1. Image Quality

Image quality (IQ) of all datasets was evaluated using a 4-point Likert scale derived from de Rotte et al. [15], rating the following three categories.

- Overall image quality;

Overall IQ regarding diagnostic value was assessed as follows: (3) good, (2) moderate, (1) poor, and (0) no diagnostic value.

- Anatomy;

The following anatomical structures were assessed separately: the border between the pituitary adenoma and the CS; the border between the anterior and posterior PG; the optic nerve differentiation; and the oculomotor/trigeminal nerve differentiation.

IQ of anatomical structures was rated as follows: (3) good, (2) moderate, (1) poor, and (0) no diagnostic value.

- Artifacts;

Pulsation, susceptibility, and motion artifacts were assessed separately.

IQ regarding artifacts was rated as follows: (3) no artifacts, (2) discrete artifacts, (1) severe artifacts, and (0) no diagnostic value.

#### 2.3.2. Qualitative Assessment

The 1.5/3 T and 7 T MRI datasets were compared regarding lesion detection (yes or no), intrasellar lesion location (right, medial, or left) and cavernous sinus infiltration (yes or no).

#### 2.3.3. Quantitative Assessment

Lesion diameters were measured in the coronal and sagittal slices with maximum lesion size in 1.5/3 T and 7 T MRI and compared.

All MRI datasets were evaluated regarding CS infiltration using a 6-point grading system derived from Knosp et al. [22] and adapted after Micko et al. [25]:

(0) Adenoma does not reach the medial internal carotid artery, no CS infiltration;
(1) Adenoma does not extend the median internal carotid artery;
(2) Adenoma extends the median internal carotid artery, but does not extend the lateral internal carotid artery;
(3a) Adenoma extends beyond the lateral internal carotid artery into the superior CS
(3b) Adenoma extends beyond the lateral internal carotid artery into the inferior CS;
(4) Adenoma completely surrounds the internal carotid artery.

**Figure 2:**
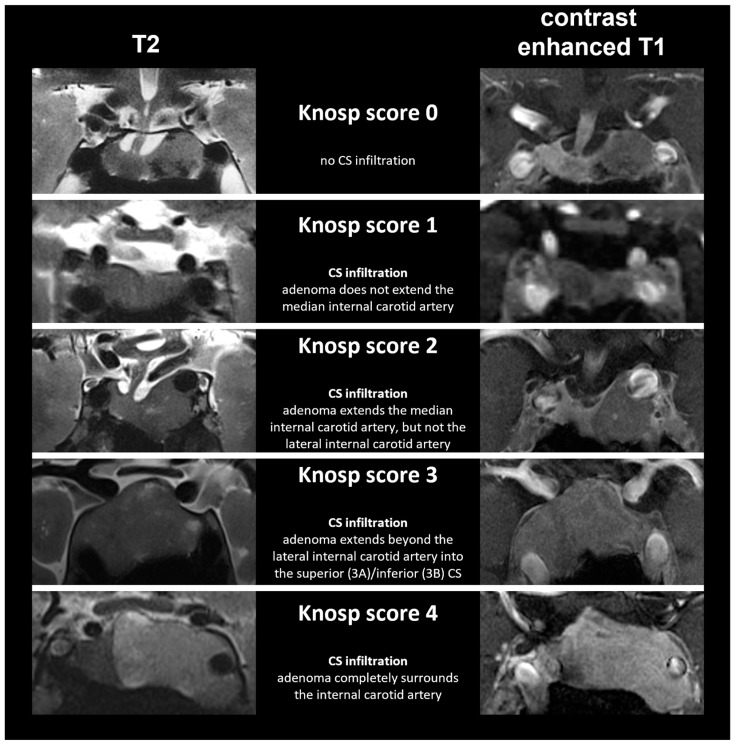
Exemplary 7 T MRI of pituitary macroadenomas infiltrating the cavernous sinus illustrating different Knosp scores in coronal view (CS = cavernous sinus).

Figure 2 presents exemplary 7 T MRI images of PMAs with CS infiltration illustrating different Knosp scores.

#### 2.3.4. Intraoperative Findings

The neurosurgeons (S.-M.S. and M.B.) intraoperatively evaluated the cavernous sinus with regard to a possible PMA infiltration (yes or no). Additionally, dural specimens were examined using routine histological methods and assessed for invasion by pituitary adenoma tissue. Intraoperative findings were compared with radiologic findings of 1.5/3 T and 7 T MRI.

### 2.4. Statistical Analysis

IQ and lesion size were analyzed using descriptive statistics. IQ values are displayed as the mean values of both reader’s assessments. IQ and lesion size were tested for normal distribution by using the Shapiro–Wilk test (if *p* > 0.05, normality was accepted). IQ parameters and lesion size were then tested for a significant difference between 1.5/3 T and 7 T MRI as follows: if IQ values showed a Gaussian distribution, an unpaired, two-tailed *t*-test was used; if IQ values showed no Gaussian distribution the Mann–Whitney U-test was used. If lesion diameters showed a Gaussian distribution, a paired, two-tailed *t*-test was used; if lesion diameters showed no Gaussian distribution, the Wilcoxon matched-pairs signed-rank test was used.

The correlation of lesion diameters between 1.5/3 T and 7 T MRI was calculated as follows: if the lesion diameters showed a Gaussian distribution, the Pearson correlation coefficients was computed; if the lesion diameters showed no Gaussian distribution, the nonparametric Spearman correlation was computed.

*p*-values less than 0.05 were considered statistically significant. *p*-values less than 0.05 are marked with *; *p*-values less than 0.01 are marked with **; *p*-values less than 0.001 are marked with ***; *p*-values less than 0.0001 are marked with ****.

Statistical analysis was performed with GraphPad Prism 9 (GraphPad Software, San Diego, USA) and Excel (Microsoft, Redmond, USA) by F.E.

## 3. Results

### 3.1. Patients

Fourteen patients with histologically proven PMA were included in our analysis (n_female_ = 7, n_male_ = 7; mean age = 60.4 years ± 16.4).

Seven PMAs were hormone-negative, three PMAs were growth-hormone-releasing, three PMAs were growth-hormone- and prolactin-releasing and one PMA was ACTH-secreting.

Nine of the included patients underwent preoperative 1.5 T MRI; five of the included patients underwent preoperative 3 T MRI.

Table 2 summarizes patient characteristics.

### 3.2. Image Quality

#### 3.2.1. Overall Image Quality

All datasets were of diagnostic value. The overall IQ of 7 T MRI datasets was rated significantly higher than the overall IQ of 1.5 T MRI datasets (IQ_overall 1.5T_ = 2.11 ± 0.60, IQ_overall 7T_ = 2.78 ± 0.44, p_IQ overall 1.5/7T_ = 0.0448). The overall IQ of 7 T MRI was also rated higher than the overall IQ of 3 T MRI datasets (IQ_overall 3T_ = 2.80 ± 0.45, IQ_overall 7T_ = 3.0 ± 0.0), however the difference was not statistically significant (p_IQ overall 3T/7T_ > 0.9999).

#### 3.2.2. Anatomy 1.5 T vs. 7 T MRI

Each evaluated anatomical parameter showed a higher IQ rating in 7 T MRI. The IQ of the optic nerve and oculomotor/trigeminal nerve differentiation was rated significantly higher in 7 T MRI (p_optic nerve differentiation 1.5/7T_ = 0.0023; p_oculomotor/trigeminal nerve differentiation 1.5/7T_ = 0.0006). No significant differences were observed in the IQ evaluation of the other anatomical structures assessed (p_IQborder PG/CS 1.5/7T_ = 0.0628; p_IQ border anterior/posterior PG 1.5/7T_ = 0.7668).

#### 3.2.3. Anatomy 3 T vs. 7 T MRI

Each evaluated anatomical parameter showed a higher or at least equal IQ rating in 7 T MRI compared with 3 T MRI. No significant differences were observed in the IQ evaluation of the anatomical structures assessed (p_IQborder PG/CS 3T/7T_ = 0.1667; p_IQ border anterior/posterior PG 3/7T_ = 0.6429; p_optic nerve differentiation 3T/7T_ > 0.9999; p_oculomotor/trigeminal nerve differentiation 3/7T_ = 0.1667).

#### 3.2.4. Artifacts 1.5 T vs. 7 T MRI

A significantly higher susceptibility for pulsation artifacts was observed in 7 T MRI compared with 1.5 T MRI (p_pulsation artifacts 1.5/7T_ = 0.0498). No significant difference was observed for susceptibility or motion artifacts (p_susceptibility artifacts 1.5/7T_ = 0.1312; p_motion artifacts 1.5T/7T_ = 0.7353).

#### 3.2.5. Artifacts 3 T vs. 7 T MRI

A significantly higher susceptibility for pulsation artifacts was observed in 7 T MRI compared with 3 T MRI (p_pulsation artifacts 3/7T_ = 0.0476). No significant difference was observed for susceptibility or motion artifacts (p_susceptibility artifacts 3/7T_ = 0.1667; p_motion artifacts 3/7T_ > 0.9999).

**Figure 3:**
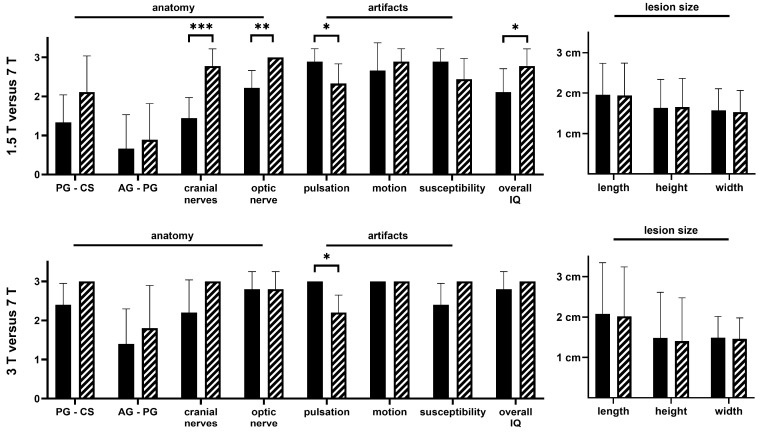
Evaluation of image quality and lesion size of 1.5 T, 3 T and 7 T sellar MRI: 7 T data are in hatched bars (PG = pituitary gland, CS = cavernous sinus, AG -PG = anterior gland-posterior gland, IQ = image quality). *p*-values less than 0.05 are marked with *; *p*-values less than 0.01 are marked with **; *p*-values less than 0.001 are marked with *** as stated in the material and methods section.

Table 3 and Figure 3 summarize the IQ evaluation between 1.5/3 T and 7 T MRI datasets.

### 3.3. Qualitative Analysis

All 1.5/3 T and 7 T MRI datasets enabled the successful identification and correct localization of all macroadenomas (n_right_ = 4; n_medial_ = 7; n_left_ = 3). Table 2 summarizes the qualitative analysis regarding lesion detection and localization.

**Figure 4:**
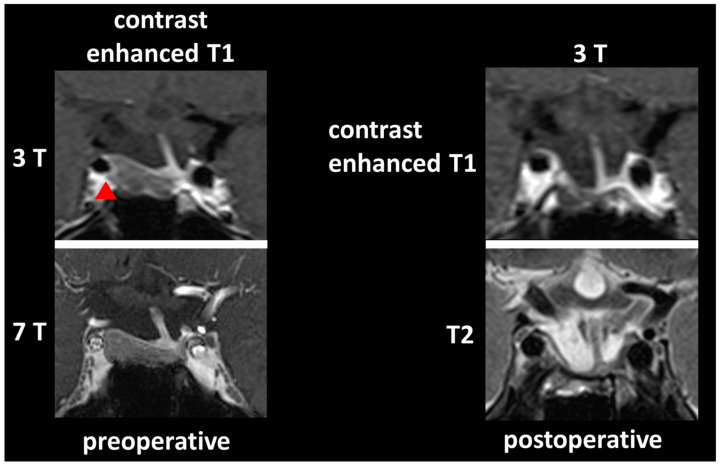
**A** 29-year-old Cushing patient with an ACTH secreting pituitary macroadenoma (contrast-spared, right-handed pituitary lesion): Knosp score was rated as “1” in preoperative 3 T MRI, which corresponds to a cavernous sinus infiltration not extending the median internal carotid artery (cavernous sinus infiltration marked with red arrowhead). In agreement with intraoperative findings, preoperative 7 T MRI showed no cavernous sinus infiltration with consequent Knosp grading “0”. Thus, complete tumor resection was achieved with consequent postoperative hormonal normalization. The 3 T MRI follow-up showed no residual tumor.

Evaluation of CS infiltration showed a different rating in two participants between 1.5/3 T and 7 T MRI: in nine 7 T MRI datasets, CS infiltration was rated with “yes”, in five 7 T MRI datasets CS infiltration was rated with “no”; in eleven 1.5/3 T MRI datasets CS infiltration was rated with “yes”, in three 1.5/3 T MRI dataset CS infiltration was rated with “no”. Figure 4 demonstrates a typical case with different rating regarding CS infiltration between 3 T and 7 T MRI.

### 3.4. Quantitative Analysis

No significant difference was observed between the evaluated macroadenoma size in 1.5/3 T and 7 T MRI (p_length 1.5/7T_ = 0.5599; p_length 3/7T_ = 0.1082; p_height1.5/7T_ = 0.3729; p_height 3/7T_ = 0.0544; p_depth 1.5/7T_ = 0.2543; p_depth 3/7T_ = 0.1778). The lesion diameters showed a strong correlation (r_length 1.5/7T_ = 0.9968, r_height 1.5/7T_ = 0.9946, r_depth 1.5/7T_ = 0.9820; r_length 3/7T_ = 0.9991, r_height 3/7T_ = 1.000, r_depth 3/7T_ = 0.9974).

Table 4 presents the mean diameters of all macroadenomas in both 1.5 T, 3 T and 7 T MRI.

Knosp score evaluation showed a different rating in two cases between 1.5/3 T and 7 T MRI: Knosp score was assessed as “1” in 1.5/3 T MRI in patient 5 and 10, whereas in 7 T MRI, Knosp score was assessed as “0” for both patients.

Table 2 contains the Knosp score evaluation for each patient in both 1.5/3 T and 7 T MRI.

### 3.5. Intraoperative Findings

The neurosurgeons reported a CS infiltration of PMA in six cases and excluded a CS infiltration in the other eight cases. Consequently, 7 T MRI showed a better agreement with intraoperative findings regarding evaluation of a CS infiltration: 7 T MRI allowed correct evaluation of CS infiltration in 78.6% of all patients (11 of 14), whereas 1.5/3 T MRI allowed correct evaluation of CS infiltration in 64.3% of all patients (9 of 14).

**Figure 5:**
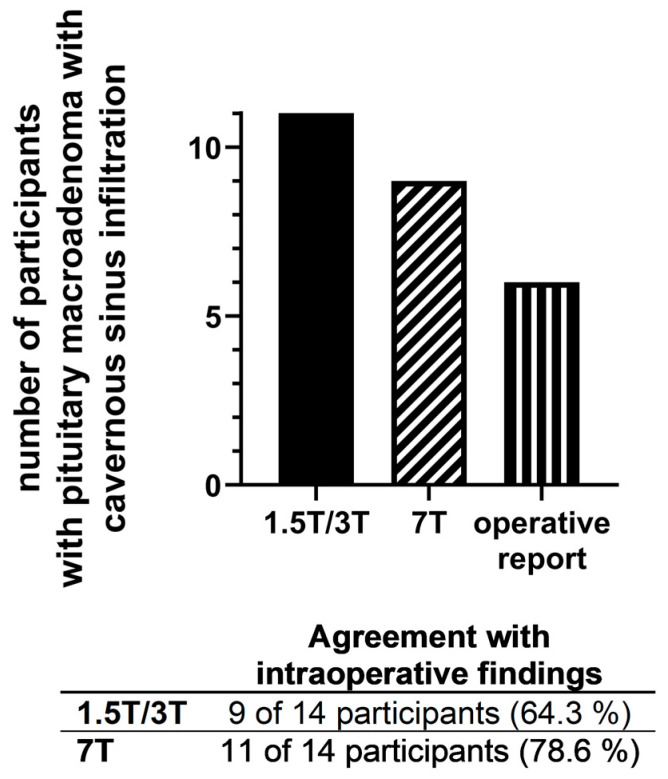
Evaluation of a cavernous sinus infiltration of the pituitary macroadenomas via 1.5 T, 3 T and 7 T MRI in comparison to intraoperative findings.

Table 2 and Figure 5 summarize evaluation of CS infiltration for 1.5/3 T and 7 T MRI compared with the intraoperative findings.

## 4. Discussion

To the best of our knowledge, this is the first study to evaluate the diagnostic power of contrast-enhanced 7 T MRI in patients with PMAs in comparison to 1.5/3 T MRI and intraoperative findings. Both standard MRI and 7 T MRI equally allowed macroadenoma detection and localization with a strong correlation regarding lesion size determination. Furthermore, overall image quality of 7 T MRI was rated better than IQ of 1.5/3 T MRI and image quality regarding all anatomical parameters of 7 T was also rated better than 1.5 T and better or at least equal to 3 T MRI. The 7 T MRI showed a higher susceptibility for pulsation artifacts than 1.5/3 T MRI; however, no significant difference was observed for susceptibility and motion artifacts. However, taking advantage of its superior spatial resolution, ultra-high-field 7 T MRI showed better agreement with intraoperative findings as the gold standard regarding the evaluation of a possible CS infiltration of PMAs: Knosp-score-based assessment of CS infiltration was correct in 78.6% of cases with 7 T MRI compared with 64.3% with lower-field strength MRI. We conclude that 7 T MR imaging is more reliable for evaluation of CS infiltration in patients with PMA.

Due to its diminutive anatomy, the surrounding osseous sella turcica and its close spatial relation to the air-filled sphenoid sinus, imaging of the PG and associated pathologies is challenging. In several studies, 3 T MRI as the current gold standard in sellar imaging is reported to be superior for the diagnosis of gland lesions, the delineation of parasellar anatomy and the detection of CS tumor infiltration compared with 1.5 T MRI [11,26]. However, following the clinical introduction of 7 T MRI, more and more studies evaluate ultra-high-field imaging of the PG: In 2016, Rotte et al. evaluated the diagnostic accuracy of 7 T MRI in patients with Cushing’s disease. The authors report additional lesion detection in 3 of their 16 patients compared with 1.5 T MRI [16]. They conclude that 7 T MRI allows a more accurate pituitary adenoma detection. In accordance, Law et al. presented a case report in which 7 T MRI enabled the detection of a pituitary lesion in a patient with Cushing’s disease with both negative 1.5 and 3 T MRI [27]. In the most recent study, Eisenhut et al. evaluated 7 T MRI for microadenoma detection and intrasellar lesion localization in comparison to 3 T MRI. In their prospective analysis, ultra-high-field MRI also surpasses 3 T MRI as it allowed the detection of three additional microadenomas in previously magnetic-resonance-negative patients [17].

However, not only patients with pituitary microadenoma could potentially benefit from preoperative 7 T MRI, but also patients with a pituitary macroadenoma—especially with regard to the identification of CS infiltration possibly limiting therapeutic success: CS infiltration which is found intraoperatively in up to 10% of all macroadenoma cases [19] aggravates surgical resection with consequently increased risk of incomplete tumor removal and higher adenoma reoccurrence rates [28,29,30]. In this context, Rutland et al. compared evaluation of Knosp scores of 7 T MRI and lower field strength MRI. The authors report significant differences between ultra-high-field imaging and 1.5/3 T MRI and conclude, that 7 T MRI allows excellent visualization of pituitary lesions and permits more accurate Knosp grading [21]. However, Rutland et al.’s work has two major drawbacks: First, all assessed datasets were non-contrast enhanced. Second, there was no comparison of the radiological determined Knosp scores to intraoperative findings as definite proof/exclusion of CS infiltration. In agreement with Rutland et al.’s conclusion, in our analysis based on contrast-enhanced datasets 7 T MRI yields a more reliable detection of tumor invasion of parasellar structures with better matching to intraoperative findings than 1.5/3 T MRI.

Thus, ultra-high-field MRI might help to improve neurosurgical resection because of its most detailed delineation of tumor boundaries allowing exact pre surgical planning and the consecutive preservation of normal pituitary gland.

In three patients there was a divergence of 7 T MRI and intraoperative findings regarding evaluation of cavernous sinus infiltration: In all three patients, a cavernous sinus infiltration of PMA has been suspected in 7 T MRI, but no cavernous sinus infiltration was intraoperatively found. In the further clinical course, two of these patients demonstrated normal pituitary hormone levels without clinical or radiologic evidence of residual or recurrent tumor. However, one of these patients suffered from rising growth hormone levels 1.5 years after surgical resection and after initial postoperative hormone normalization. The performed MRI demonstrated a recurrent pituitary tumor in the right cavernous sinus. Therefore, thorough neurosurgical intraoperative exploration of the cavernous sinus is mandatory, if a cavernous sinus infiltration is radiologically suspected.

In general, more and more imaging sites use ultra-high-field MRI scanners in the clinical routine as more and more studies demonstrate its diagnostic advantage over standard 1.5 and 3 T MRI [31]. In this context, further hard- and software optimization will help to overcome current limitations of 7 T MRI, e.g., longer scan times, and allow the transfer of research results into the clinical practice.

This analysis has some limitations. First, the low number of included macroadenoma patients. However, even in our small series, 7 T MRI demonstrated its favorable diagnostic value in PG imaging. Second, the increased susceptibility of 7 T MRI for pulsation artifacts: Because of the close spatial relation of the PG to the internal carotid artery, these artifacts could potentially complicate radiological assessment of the sellar region. However, overall IQ of 7 T MRI was rated higher than IQ of 1.5 T and 3 T MRI. In addition, prospective sequence optimization might further improve visualization of the PG and associated lesions as well as reduce the susceptibility of 7 T MRI for pulsation artifacts.

## 5. Conclusions

7 T MRI leads to a more reliable assessment of parasellar expansion of pituitary macroadenomas with better agreement to intraoperatively determined CS invasion than standard MRI. Thus, ultra-high-field 7 T MRI can improve surgical planning and support complete tumor resection.

## Figures and Tables

**Table 1:** Selected MRI sequence parameters for 1.5 T, 3 T and 7 T MRI.

	1.5 T		3 T		7 T	
	T1 cor	T2 cor	T1 cor	T2 cor	T1 cor	T2 cor
TE (ms)	9.8	88	15	93	3.18	91
TR (ms)	374	2910	484	3100	188	3380
flip angle (degree)	150	143	180	93	66	120
FOV (mm)	180	185	180	205	150	180
matrix	256 × 128	256 × 256	240 × 320	448 × 448	544 × 544	512 × 512
slice thickness (mm)	1.5	1.5	1.5	1.5	1.5	1.5
voxel size (mm × mm × mm)	0.7 × 0.7 × 1.5	0.4 × 0.4 × 1.5	0.75 × 0.56 × 1.5	0.46 × 0.46 × 1.5	0.3 × 0.3 × 1.5	0.35 × 0.35 × 1.5
acquisition time (min:sec)	2:26	2:47	3:08	5:21	3:06	3:55

cor = coronal; FOV = field of view.

**Table 2:** Patient data.

**Patients with Preoperative 1.5 T and 7 T MRI**									
			**Lesion Location**		**Knosp Score**				
**Nr.**	**Sex**	**Age**	**1.5 T**	**7 T**	**1.5 T**	**7 T**	**Intraoperative Findings**	**Hormone Status**	**Lesion Diameter** **(mm × mm × mm)**
1	female	46	left	left	4	4	CS infiltration	hormone-negative	25 × 12 × 15
2	female	61	left	left	1	1	CS infiltration	growth-hormone-releasing	15 × 12 × 11
3	female	67	medial	medial	2	2	no CS infiltration	hormone-negative	14 × 14 × 13
4	male	55	right	right	1	1	no CS infiltration	growth-hormone- and prolactin-releasing	12 × 10 × 10
5	male	56	right	right	1	0	no CS infiltration	growth-hormone- and prolactin-releasing	13 × 10 × 10
6	male	59	medial	medial	3a	3a	CS infiltration	hormone-negative	30 × 29 × 27
7	male	82	medial	medial	3a	3a	CS infiltration	hormone-negative	26 × 25 × 15
8	male	85	medial	medial	0	0	no CS infiltration	hormone-negative	22 × 21 × 13
9	male	85	right	right	3a	3a	CS infiltration	hormone-negative	26 × 21 × 16
**Patients with Preoperative 3 T and 7 T MRI**									
			**Lesion Location**		**Knosp Score**				
**Nr.**	**Sex**	**Age**	**3 T**	**7 T**	**3 T**	**7 T**	**Intraoperative Findings**	**Hormone Status**	**Lesion Diameter** **(mm × mm × mm)**
10	female	29	right	right	1	0	no CS infiltration	ACTH secreting	15 × 10 × 11
11	female	50	medial	medial	2	2	no CS infiltration	growth-hormone-releasing	19 × 19 × 17
12	female	61	left	left	0	0	no CS infiltration	growth-hormone- and prolactin-releasing	13 × 11 × 10
13	female	61	medial	medial	0	0	no CS infiltration	growth-hormone-releasing	14 × 13 × 10
14	male	35	medial	medial	3a	3a	CS infiltration	hormone-negative	43 × 33 × 23

CS = cavernous sinus; ACTH = adrenocorticotropic hormone.

**Table 3:** Image quality evaluation of 1.5 T, 3 T and 7 T sellar MRI.

	1.5 T	7 T	*p*	3 T	7 T	*p*
**overall IQ**	2.11 ± 0.60	2.78 ± 0.44	0.0448 (*)	2.80 ± 0.45	3.0 ± 0.0	>0.9999
***anatomy***						
**border between the PG and the CS**	1.33 ± 0.71	2.11 ± 0.93	0.0628	2.40 ± 0.55	3.00 ± 0.0	0.1667
**border between anterior and posterior PG**	0.67 ± 0.87	0.89 ± 0.93	0.7668	1.40 ± 0.89	1.80 ± 1.10	0.6429
**optic nerve differentiation**	2.22 ± 0.44	3.00 ± 0.0	0.0023 (**)	2.80 ± 0.45	2.80 ± 0.45	>0.9999
**oculomotor/trigeminal nerve differentiation**	1.44 ± 0.53	2.78 ± 0.44	0.0006 (***)	2.20 ± 0.84	3.00 ± 0.0	0.1667
***artifacts***						
**pulsation artifacts**	2.89 ± 0.33	2.33 ± 0.5	0.0498 (*)	3.00 ± 0.0	2.20 ± 0.45	0.0476 (*)
**susceptibility artifacts**	2.89 ± 0.33	2.44 ± 0.53	0.1312	2.40 ± 0.55	3.00 ± 0.0	0.1667
**motion artifacts**	2.67 ± 0.71	2.89 ± 0.33	0.7353	3.00 ± 0.0	3.00 ± 0.0	>0.9999

IQ = image quality; CS = cavernous sinus; PG = pituitary gland. *p*-values less than 0.05 are marked with *; *p*-values less than 0.01 are marked with **; *p*-values less than 0.001 are marked with *** as stated in the material and methods section.

**Table 4:** Pituitary macroadenoma size evaluation in 1.5 T, 3 T and 7 T MRI.

	1.5 T	7 T	*p*	r	3 T	7 T	*p*	r
**length_mean_ (cm)**	1.96 ± 0.78	1.94 ± 0.80	0.5599	0.9968	2.08 ± 1.27	2.02 ± 1.22	0.1082	0.9991
**height_mean_ (cm)**	1.63 ± 0.71	1.65 ± 0.71	0.3729	0.9946	1.48 ± 1.13	1.40 ± 1.07	0.0544	1.000
**depth_mean_ (cm)**	1.58 ± 0.53	1.53 ± 0.53	0.2543	0.9820	1.49 ± 0.52	1.46 ± 0.52	0.1778	0.9974

## Data Availability

The data presented in this study are available on request from the corresponding author.
